# A novel nomogram incorporating preoperative systemic inflammatory response index and clinicopathological parameters for predicting lymph node metastasis in endometrial cancer

**DOI:** 10.3389/fonc.2026.1862350

**Published:** 2026-07-07

**Authors:** Youlin Deng, Xiuling Shi, Chunxia Gong, Hangkun Yu, Lamei Hou, Zhuoying Hu, Peng Jiang

**Affiliations:** 1 Department of Obstetrics and Gynecology, Chongqing Key Laboratory of Maternal and Fetal Medicine, The First Affiliated Hospital of Chongqing Medical University, Chongqing, China; 2 Department of Obstetrics and Gynecology, Women and Children's Hospital of Chongqing Medical University, Chongqing, China; 3 Department of Gynecology, Fengdu People's Hospital, Chongqing, China

**Keywords:** endometrial cancer, lymph node metastasis, nomogram, prediction, systemic inflammatory response index

## Abstract

**Objective:**

This study aimed to evaluate whether preoperative systemic inflammatory response index (SIRI) could predict lymph node metastasis (LNM) in endometrial cancer (EC), and to develop a nomogram that combines SIRI with clinicopathological parameters for individualized LNM risk assessment.

**Methods:**

We retrospectively enrolled 1, 336 EC patients who underwent primary surgery. Among them, 947 cases from the First Affiliated Hospital of Chongqing Medical University served as the training cohort, and 389 cases from the Chongqing Maternal and Child Health Hospital comprised the external validation cohort. Preoperative SIRI was calculated using peripheral neutrophil, monocyte, and lymphocyte counts. Logistic regression analyses were used to identify independent predictors of LNM, which were then incorporated into a nomogram. Model performance was evaluated by ROC curves and calibration curves in both cohorts. Kaplan-Meier analysis was applied to compare recurrence-free survival (RFS) and overall survival (OS) between high- and low-risk groups stratified by the model.

**Results:**

SIRI yielded an area under the curve (AUC) of 0.773 for predicting LNM, with a sensitivity of 74.2% and specificity of 75.8%. The optimal cutoff value of SIRI was 1.115. Multivariate analysis showed that age, CA125, histological type, molecular classification, and SIRI were independently associated with LNM (P < 0.001). A nomogram integrating these five factors was then constructed. This combined model achieved AUC of 0.889 in the training cohort, outperforming the models based solely on SIRI (AUC = 0.750) or solely on clinicopathological parameters (AUC = 0.836). Calibration curves indicated good agreement between predictions and observations. Using a cutoff of 0.136, we divided patients into high- and low-risk groups and found marked differences in both RFS and OS between training and validation cohorts (P < 0.05).

**Conclusion:**

Preoperative SIRI is an independent predictor of LNM in patients with EC. The nomogram model incorporating SIRI and clinicopathological parameters demonstrated superior performance in predicting LNM compared to models containing either SIRI alone or only traditional parameters, providing a valuable tool for individualized preoperative risk stratification and surgical decision-making.

## Introduction

Endometrial cancer (EC) is one of the most common gynecologic malignancies worldwide (1). Globally, an estimated 420, 242 new cases and 97, 704 deaths were reported in 2022 (2). Its incidence continues to rise, largely driven by increasing obesity rates and an aging demographic. Lymph node metastasis (LNM) represents one of the most powerful prognostic factors in EC, and is strongly associated with unfavorable survival and an elevated risk of recurrence (3). Even minimal nodal involvement, including micrometastases, is associated with unfavorable outcomes (4). Consequently, accurate preoperative identification of LNM is critical for surgical planning and adjuvant therapy decisions.

However, systematic lymphadenectomy, the conventional approach for nodal staging, is associated with substantial morbidity, including prolonged operative time, increased blood loss, and postoperative lymphedema (5). Evidence from prospective randomized trials indicates that complete lymphadenectomy offers no survival advantage for low-risk patients, implying that many undergo unnecessary extensive surgery (6). These observations underscore the urgent need to pinpoint high-risk patients who genuinely require thorough nodal assessment while protecting low-risk individuals from avoidable procedures. Existing predictive tools, which rely on conventional clinicopathological factors like tumor grade, myometrial invasion depth, and serum CA125 levels, suffer from notable heterogeneity and a lack of independent external validation (7). Thus, novel, readily obtainable biomarkers are urgently needed to enhance preoperative risk stratification. In this context, systemic inflammation-based indices have recently garnered attention as promising prognostic markers across various malignancies (8–10).

Chronic inflammation is recognized as a hallmark of cancer, contributing to tumor initiation, progression, metastasis, and therapy resistance (11). Derived from routine complete blood counts, systemic inflammatory indices—including the neutrophil-to-lymphocyte ratio (NLR), platelet-to-lymphocyte ratio (PLR), lymphocyte-to-monocyte ratio (LMR), systemic immune-inflammation index (SII), and systemic inflammation response index (SIRI)—have been extensively explored as prognostic biomarkers in multiple cancer types (12). SIRI, defined as (neutrophil count × monocyte count)/lymphocyte count, captures the equilibrium between pro-tumor inflammatory cells (neutrophils and monocytes) and anti-tumor immune cells (lymphocytes). Elevated SIRI has been linked to poor survival outcomes in esophageal, gastric, breast, and cervical cancers (13, 14).

With respect to EC, it is noteworthy that the majority of previous studies have focused on the prognostic value of postoperative inflammatory indices. However, postoperative indices are susceptible to multiple confounding factors, including surgical trauma, anesthesia, and perioperative interventions. Moreover, they cannot inform clinical decision-making prior to treatment, thereby offering limited value for guiding preoperative individualized management. In contrast, multiple studies have reported associations between elevated preoperative NLR, PLR, or SII and deeper myometrial invasion, advanced FIGO stage, and LNM (15). Nevertheless, the precise role of SIRI in EC remains inadequately characterized. Although one retrospective study has shown a relationship between SIRI and EC prognosis (16), the utility of preoperative SIRI—particularly when combined with traditional clinicopathological parameters—for preoperative prediction of LNM has yet to be systematically validated. Given that SIRI integrates three distinct inflammatory cell lineages, it may offer a more comprehensive picture of systemic inflammation than single-ratio indices, meriting further investigation in the context of EC nodal metastasis.

Therefore, this study sought to evaluate the predictive value of preoperative SIRI for LNM in EC patients and to construct a combined predictive model integrating preoperative SIRI with conventional clinicopathological parameters. We further assessed the discriminative performance of this combined model and verified its clinical applicability for preoperative risk stratification. By accurately identifying patients at elevated risk for LNM, this model could facilitate individualized surgical decision-making, reduce unnecessary lymphadenectomy in low-risk patients, and ultimately support a more personalized, risk-adapted strategy for EC management.

## Materials and methods

### Study population

**Figure 1:**
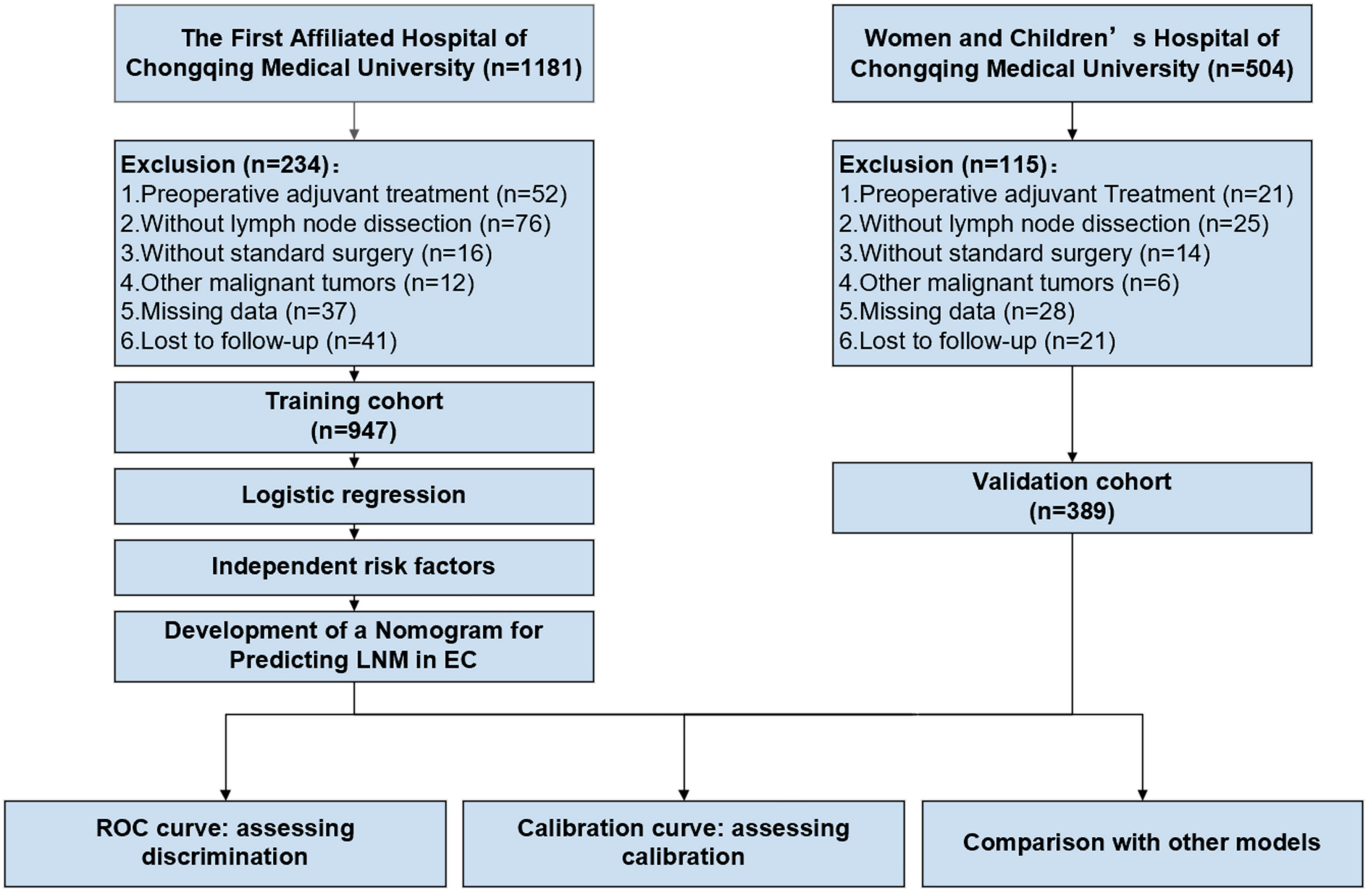
Flowchart of patient selection and cohort allocation.

This retrospective study enrolled patients with FIGO 2009 stage I–III EC (17) who underwent surgical treatment at two hospitals in China between 2018 and 2022. The development cohort comprised 947 cases from the First Affiliated Hospital of Chongqing Medical University, and the external validation cohort included 389 cases from the Women’s and Children’s Hospital of Chongqing Medical University. Exclusion criteria were: receipt of neoadjuvant therapy; no lymphadenectomy; non-standard surgery; other concurrent malignancies; incomplete clinicopathological data; and loss to follow-up. The study design flowchart is presented in **Figure 1**.

### Data collection

Data were collected by uniformly trained investigators using standardized case report forms, encompassing clinicopathological characteristics, preoperative laboratory findings, and surgical information. Preoperative blood samples were obtained within one week prior to surgery. Variables included demographic and clinical parameters (age, body mass index (18), dichotomized as <24 and ≥24 kg/m² according to the Chinese definition of overweight (19), serum CA125 level, histological type, and molecular classification) and preoperative SIRI. Surgical parameters (scope of lymphadenectomy and number of LN removed) were recorded for descriptive purposes, whereas LNM status served as the outcome variable. Only preoperatively available variables were included as predictors. Variables requiring postoperative pathological assessment (e.g., LVSI, myometrial invasion depth, tumor grade) were not incorporated into the model. The SIRI was calculated as neutrophil count × monocyte count/lymphocyte count and categorized into high and low groups based on the optimal cutoff value. Grade 1–2 endometrioid adenocarcinoma was classified as type I (estrogen-dependent), while grade 3 and non-endometrioid histologies were classified as type II (non-estrogen-dependent) (20).

### Histology and immunohistochemistry

Immunohistochemical analysis was conducted on formalin-fixed, paraffin-embedded tissue sections. p53 expression was evaluated using an anti-p53 antibody (60283-2-Ig, 1:2000), with staining patterns categorized as either normal-defined as heterogeneous nuclear staining in 1%–80% of tumor cells-or abnormal, comprising either complete loss of nuclear staining or strong diffuse nuclear positivity in ≥80% of tumor cells, as previously described (21). Mismatch repair status was assessed through immunohistochemical staining of four key proteins: MSH2, MSH6, PMS2, and MLH1, using anti-MSH2 (82775-1-RR, 1:2000), anti-MSH6 (18120-1-AP, 1:2000), anti-PMS2 (68905-5-Ig, 1:2000), and anti-MLH1 (84208-2-RR, 1:300) antibodies. Complete absence of nuclear expression in any single protein was classified as mismatch repair-deficient (dMMR), whereas retained expression of all four proteins indicated mismatch repair-proficient (pMMR) status (22). Given the retrospective study design and the lack of insurance reimbursement for POLE hotspot mutation testing during the study period, this assay was only available for 108 (11.4%) and 26 (6.7%) patients in the training and validation cohorts, respectively; the remaining patients lacked this information. Consequently, molecular subtyping followed a sequential classification algorithm based on combined MMR and p53 status, yielding three distinct subgroups: dMMR, p53 abnormal (abn), and pMMR/p53 wild-type (wt) (23).

### Surgical treatment and follow-up

All patients underwent comprehensive surgical staging, consisting of total hysterectomy, bilateral salpingo-oophorectomy, and systematic pelvic LN dissection (PLND). Sentinel lymph node (SLN) biopsy and/or para-aortic LN dissection were performed selectively based on indications. Patients with high-risk features—grade 3 EC, deep myometrial invasion, or pelvic SLN metastasis identified intraoperatively or postoperatively—underwent extended dissection to the para-aortic region (24). Adequacy of lymphadenectomy followed the criteria proposed by AlHilli et al.: ≥10 pelvic nodes; if para-aortic dissection was performed, ≥5 nodes (25).

Postoperative adjuvant therapy (radiotherapy alone or concurrent chemoradiotherapy) was individualized following international guidelines and multidisciplinary discussion (23). Follow-up commenced from the date of surgery and continued through March 2026: every 3 months for the first 2 years, every 6 months for years 3–5, and annually thereafter. Evaluations included physical examination, laboratory tests, imaging studies, and histological biopsy when indicated. Recurrence was defined as new lesions at the vaginal cuff, pelvis, peritoneum, or distant sites, with histological confirmation whenever feasible (20, 26). Recurrence-free survival (RFS) was measured from complete tumor resection to the first occurrence of recurrence (histologically or radiologically confirmed) or death from any cause; patients without events were censored at last follow-up (27). Overall survival (OS) was measured from primary surgery to death from any cause, with survivors censored at last follow-up (27).

### Statistical analysis

Continuous variables were presented as median (interquartile range) or mean ± standard deviation, and compared using Student’s t-test or Mann-Whitney U test as appropriate. Categorical variables were expressed as frequencies (percentages) and analyzed using χ² test or Fisher’s exact test. Univariate and multivariate logistic regression analyses were performed to identify independent predictors of LNM, with results reported as odds ratios (OR) and 95% confidence intervals (CI). Variables with P < 0.05 in univariate analysis were entered into multivariate analysis.

A predictive nomogram was constructed based on multivariate logistic regression coefficients. The discriminative performance was evaluated using the area under the receiver operating characteristic curve (AUC), with comparison between models by DeLong’s test. Calibration was assessed using calibration curves. The optimal probability cutoff was determined by the Youden index (sensitivity + specificity − 1) (28). Kaplan-Meier method with log-rank test was used for survival analysis. All statistical analyses were performed using R software (version 4.2.1) or SPSS (version 26.0), with two-sided P < 0.05 considered statistically significant.

## Results

### Patient characteristics

As shown in **Figure 1** and **Table 1**, the training cohort and validation cohort included 947 and 389 patients with EC, respectively. The two groups were balanced in major clinicopathological characteristics, including age, BMI, CA125, histotype, molecular classification, SIRI, extent and number of LN dissections, and number of LNM, with no significant differences observed (all P > 0.05), indicating good comparability between the cohorts. In the training cohort, the mean age of patients was 53.77 years, with a mean BMI of 24.46. A total of 74.8% of patients had CA125 < 35 U/ml, 73.3% were classified as Type I histology, 24.8% were p53 abn, 68.4% underwent only pelvic LN dissection, and 84.1% had no LNM. In the validation cohort, the mean age was 52.97 years, with a mean BMI of 24.61. A total of 71.5% of patients had CA125 < 35 U/ml, 75.6% were Type I histology, 23.9% were p53 abn, 66.1% underwent only pelvic LN dissection, and 87.4% had no LNM.

**Table 1:** Demographic and clinical characteristics of patients with EC.

Characteristic	Training cohort (n=947)	Validation cohort (n=389)	P-value
Age (years)			0.172
Mean ± SD	53.77(± 9.53)	52.97(± 10.15)	
BMI (kg/m^2^)			0.499
Mean ± SD	24.46(± 3.69)	24.61(± 3.72)	
CA125 (U/ml)			0.213
< 35	708(74.8%)	278(71.5%)	
≥ 35	239(25.2%)	111(28.5%)	
Histotype			0.385
Type I	694(73.3%)	294(75.6%)	
Type II	253(26.7%)	95(24.4%)	
Molecular classification			0.068
pMMR-p53 wt	467(49.3%)	216(55.5%)	
dMMR	245(25.9%)	80(20.6%)	
p53 abn	235(24.8%)	93(23.9%)	
SIRI			0.707
Median (P25, P75)	0.75[0.51, 1.32]	0.74[0.51, 1.32]	
Scope of lymphadenectomy			0.402
Only pelvic LNs	648(68.4%)	257(66.1%)	
Pelvic + Para-aortic LNs	299(31.6%)	132(33.9%)	
Number of LNs removed			
Only pelvic LNs Median	30[22, 37]	29[21, 37]	0.402
Para-aortic LNs Median	10[6, 15]	11[6, 16]	0.309
LNM			0.297
None	796(84.1%)	340(87.4%)	
Only pelvic LNM	129(13.6%)	42(10.8%)	
Pelvic + Para-aortic LNM	22(2.3%)	7(1.8%)	
Death			0.280
No	859(90.7%)	360(92.5%)	
Yes	88(9.3%)	29(7.5%)	
Follow-up (months)			0.795
Median (P25, P75)	47[39, 62]	46[40, 60]	

BMI, body mass index; CA125, cancer antigen 125; EC, endometrial cancer; LN, lymph node; LNM, lymph node metastasis; P25, 25th percentile; P75, 75th percentile; SD, standard deviation; SIRI, systemic inflammatory response index.

### Prognostic value of SIRI in EC

**Figure 2:**
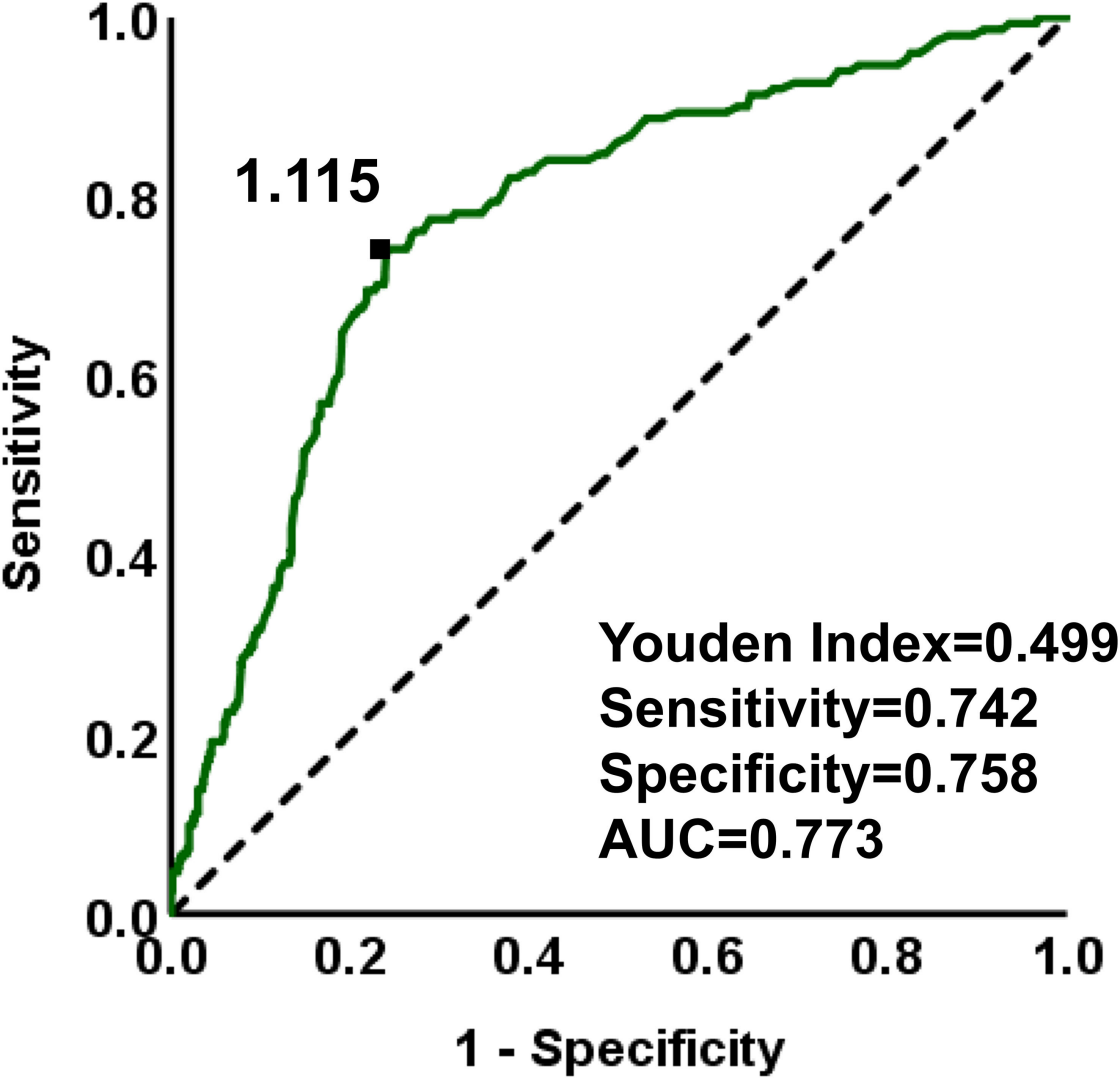
ROC curve analysis of SIRI for predicting LNM in the training cohort. The optimal cutoff value was determined to be 1.115 (AUC = 0.773, sensitivity = 74.2%, specificity = 75.8%).

ROC curve analysis revealed that in the training cohort, the AUC of SIRI for predicting LNM in EC was 0.773, with a sensitivity of 74.2% and a specificity of 75.8%. Based on the ROC curve and Youden index, the optimal cutoff value of SIRI for predicting LNM was further determined to be 1.115, by which patients in the training cohort were divided into high-SIRI and low-SIRI groups (**Figure 2**). Comparison between high-SIRI and low-SIRI patients in the training cohort showed no significant differences in age and BMI, whereas CA125, histological type, and molecular classification were statistically significant (P < 0.05, **Table 2**). These findings were similarly validated in the validation cohort (**Table 2**).

**Table 2:** Comparison of clinicopathological characteristics by SIRI grouping in the training cohort.

Characteristic	Training cohort			Validation cohort		
	SIRI≥1.115 (n=305)	SIRI<1.115 (n=642)	P-value	SIRI≥1.115 (n=123)	SIRI<1.115 (n=266)	P-value
Age (years)	53.88(± 9.82)	53.72(± 9.39)	0.920	53.04(± 10.73)	52.94(± 9.89)	0.897
BMI (kg/m^2^)	27.10(± 4.10)	24.35(± 3.47)	0.891	24.75(± 4.13)	24.55(± 3.53)	0.685
CA125 (U/ml)			<0.001			0.024
< 35	209(68.5%)	499(77.7%)		83(67.5%)	208(78.2%)	
≥ 35	96(31.5%)	143(22.3%)		40(32.5%)	58(21.8%)	
Histotype			<0.001			0.019
Type I	195(63.9%)	499(77.7%)		82(66.7%)	207(77.8%)	
Type II	110(36.1%)	143(22.3%)		41(33.3%)	59(22.2%)	
Molecular classification			0.002			0.013
pMMR-p53 wt	128(42.0%)	339(52.8%)		51(41.5%)	153(57.5%)	
dMMR	82(26.9.0%)	163(25.4%)		32(26.0%)	52(19.5%)	
p53 abn	95(31.1%)	140(21.8%)		40(32.5%)	61(22.9%)	

BMI, body mass index; CA125, cancer antigen 125; dMMR, deficient mismatch repair; p53 abn, p53 abnormal; p53 wt, p53 wild-type; pMMR, proficient mismatch repair; SIRI, systemic inflammatory response index.

### Independent risk factors for LNM

**Figure 3:**
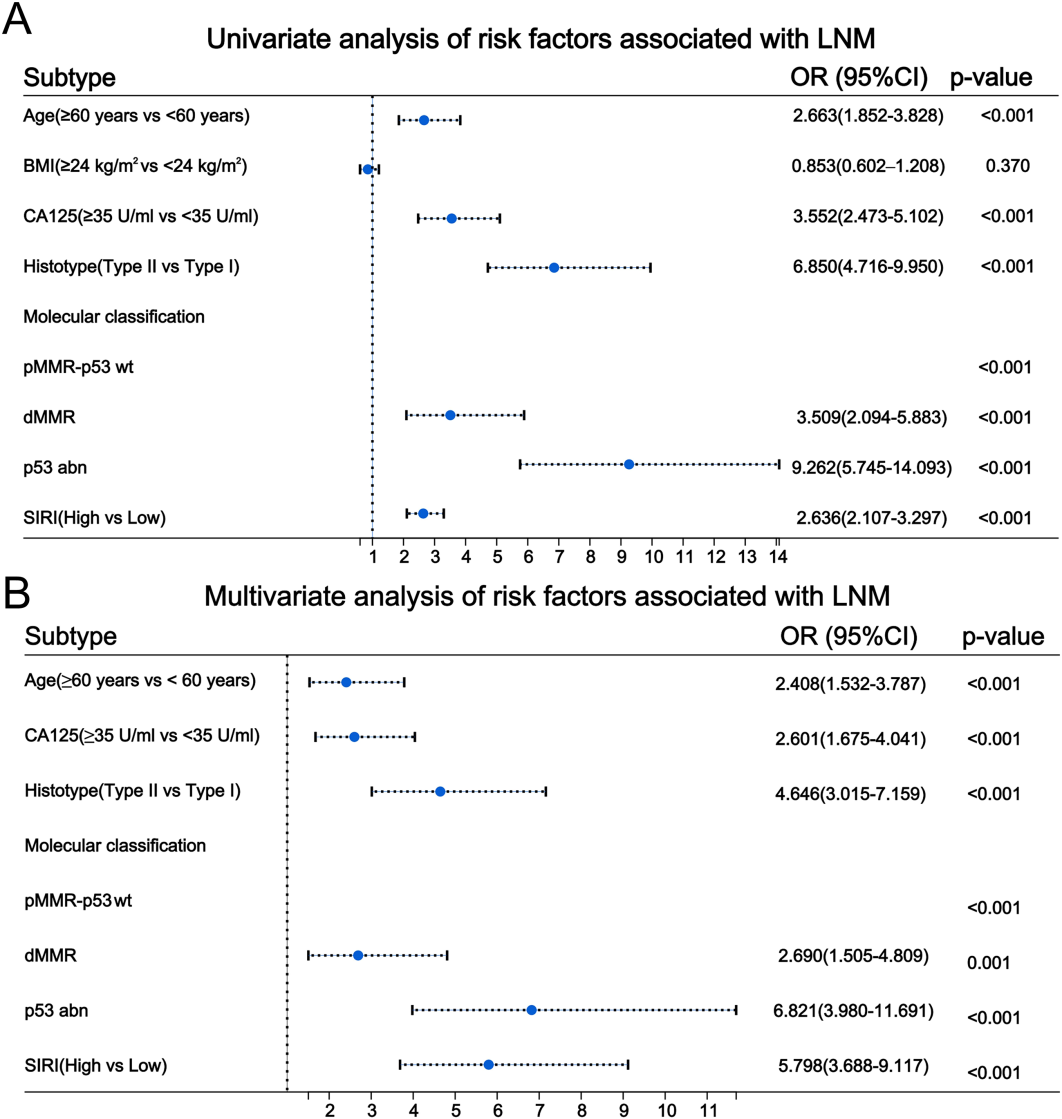
Forest plots of univariate **(A)** and multivariate **(B)** logistic regression analyses for LNM prediction.

Univariate logistic regression analysis revealed that age (OR = 2.663, P < 0.001), CA125 (OR = 3.552, P < 0.001), histological type (OR = 6.850, P < 0.001), molecular classification (P < 0.001), and SIRI (OR = 2.636, P < 0.001) were significantly associated with LNM. Conversely, BMI showed no significant association (OR = 0.853, P = 0.370). Variables demonstrating statistically significant associations in univariate analysis (P < 0.05) were subsequently entered into the multivariate logistic regression model (**Figure 3A**; **Table 3**). Multivariate logistic regression analysis identified five independent prognostic factors: age (OR = 2.408, P < 0.001), CA125 (OR = 2.601, P < 0.001), histological type (OR = 4.646, P < 0.001), molecular classification (OR = 6.821, P < 0.001), and SIRI (OR = 5.798, P < 0.001) (**Figure 3B**; **Table 3**). A nomogram for preoperative prediction of LNM was subsequently developed incorporating these five independent predictors.

**Table 3:** Univariate and multivariate binary logistic regression analysis of predictors for LNM in EC.

Variables	Univariate analysis			Multivariate analysis		
	OR	95% CI	p-value	OR	95% CI	p-value
Age (≥60 years vs <60 years)	2.663	1.852-3.828	<0.001	2.408	1.532-3.787	<0.001
BMI (≥24kg/m2 vs <24 kg/m2)	0.853	0.602-1.208	0.370	–	–	–
CA125 (≥ 35 U/ml vs <35 U/ml)	3.552	2.473-5.102	<0.001	2.601	1.675-4.041	<0.001
Histotype (Type II vs Type I)	6.850	4.716-9.950	<0.001	4.646	3.015-7.159	<0.001
Molecular classification						
pMMR-p53 wt	–	–	<0.001	–	–	<0.001
dMMR	3.509	2.094-5.883	<0.001	2.690	1.505-4.809	0.001
p53 abn	9.262	5.745-14.093	<0.001	6.821	3.980-11.691	<0.001
SIRI (≥1.115 vs <1.115)	2.636	2.107-3.297	<0.001	5.798	3.688-9.117	<0.001

abn, abnormal; BMI, body mass index; CI, confidence interval; dMMR, deficient mismatch repair; EC, endometrial cancer; LNM, lymph node metastasis; OR, odds ratio; p53 abn, p53 abnormal; p53 wt, p53 wild-type; pMMR, proficient mismatch repair; SIRI, systemic inflammation response index; wt, wild-type.

### The establishment and validation of prediction model for LNM

**Figure 4:**
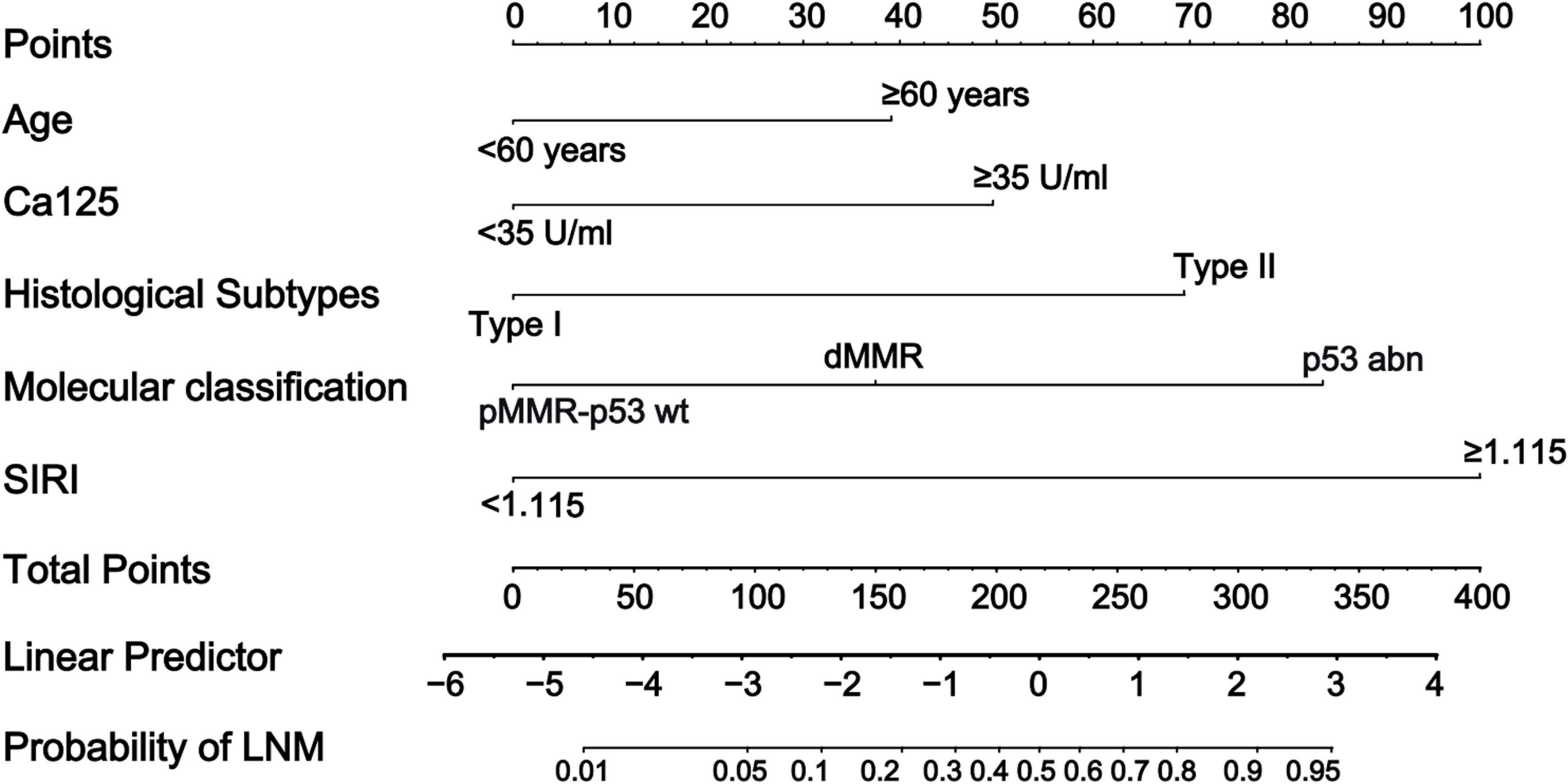
Nomogram model for predicting LNM in EC patients. The model incorporates age, CA125, histological type, molecular classification, and SIRI.

**Figure 5:**
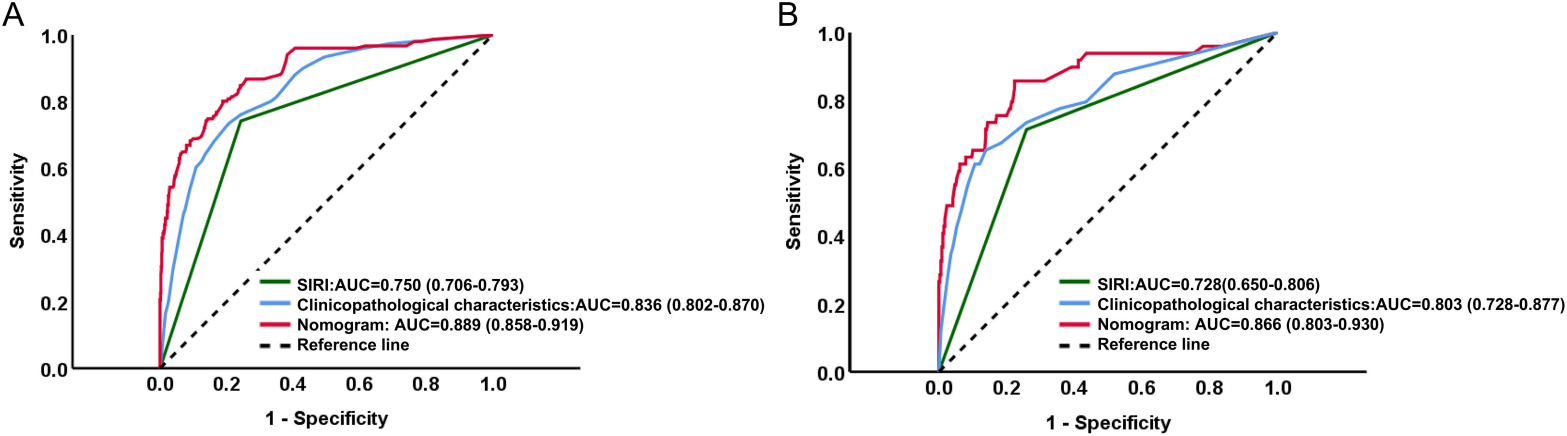
ROC curves comparing the predictive performance of different models in the training cohort **(A)** and validation cohort **(B)**.

**Figure 6:**
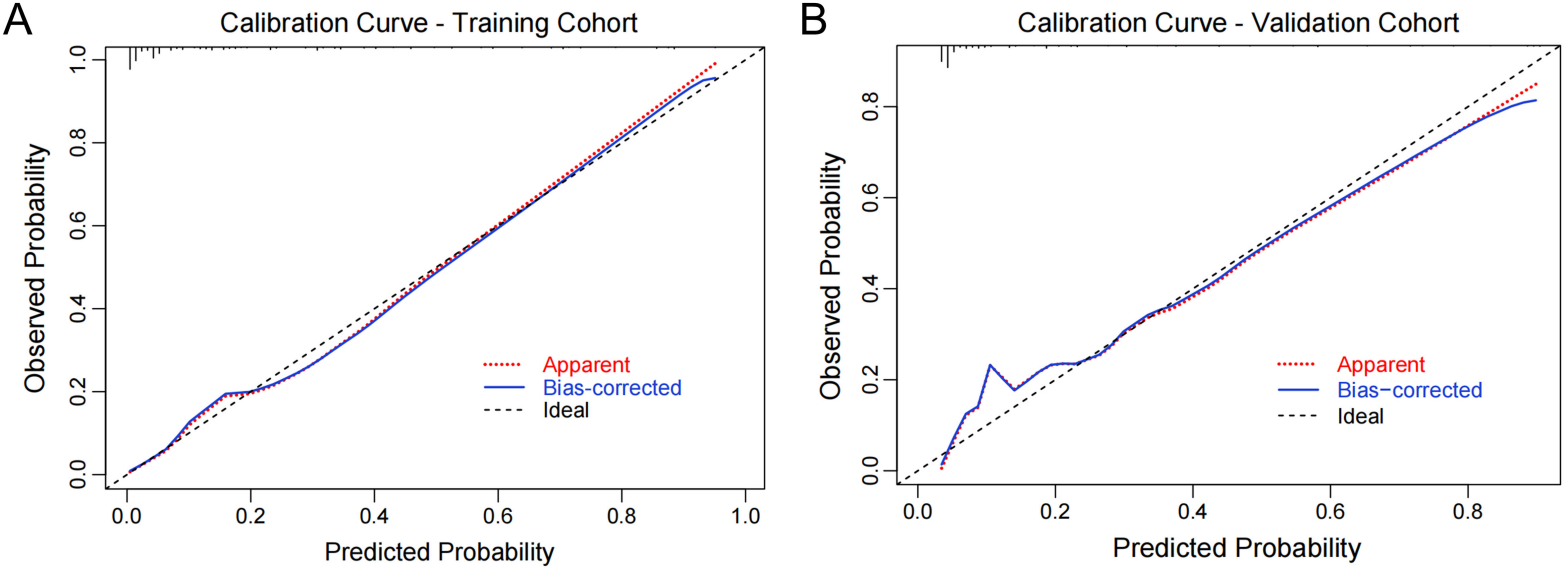
Calibration curves of the nomogram in the training cohort **(A)** and validation cohort **(B)**, showing good agreement between predicted and observed probabilities of LNM.

The five independent prognostic factors including age, CA125, histological type, molecular classification, and SIRI were used to construct a nomogram model (**Figure 4**). This nomogram demonstrated the contribution of each variable to the risk of LNM, where the length of the line segment represents the weight of the corresponding predictor. As shown in the figure, SIRI corresponded to a longer line segment even compared with other clinicopathological factors, indicating its significant predictive value in predicting LNM in EC. The ROC curve indicated that the model constructed by combining SIRI with clinicopathological factors (AUC = 0.889) exhibited stronger predictive performance, which was significantly superior to the model using SIRI alone (AUC = 0.750) or preoperative clinicopathological parameters alone (AUC = 0.836) in the training cohort (**Figure 5A**). Consistent results were observed in the validation cohort, with the combined model achieving an AUC of 0.866, compared to 0.728 for SIRI alone and 0.803 for clinicopathological parameters alone (**Figure 5B**). To evaluate the predictive accuracy of the nomogram model, internal and external validations were performed. The calibration curve revealed a high consistency between the model-predicted probability of LNM and the actually observed LNM, suggesting that the model had favorable accuracy (**Figure 6**).

### Risk stratification and prediction performance

**Figure 7:**
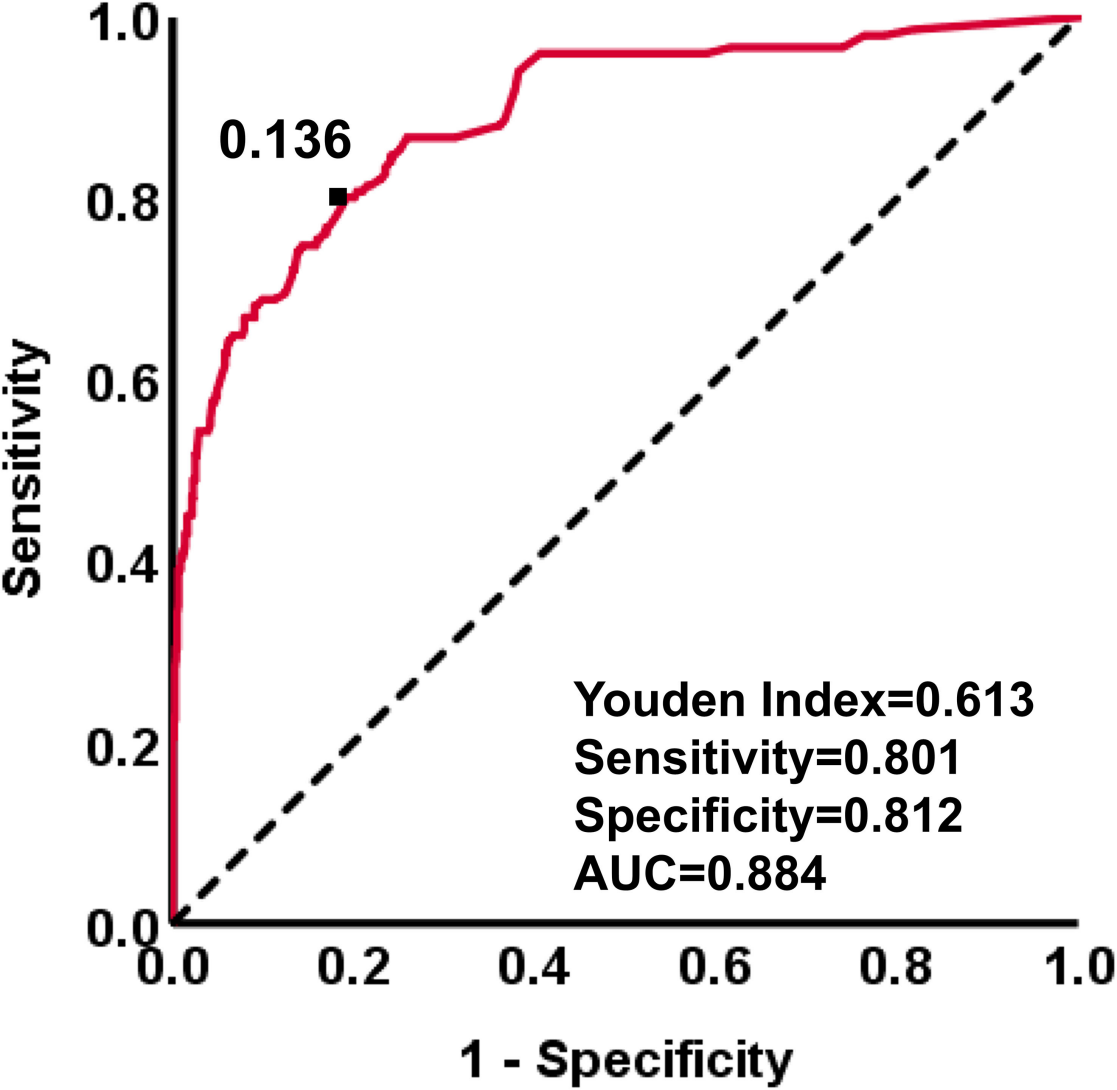
ROC curve analysis for determining the optimal cutoff value (0.136) of the nomogram for risk stratification (AUC = 0.884, sensitivity = 80.1%, specificity = 81.2%).

**Figure 8:**
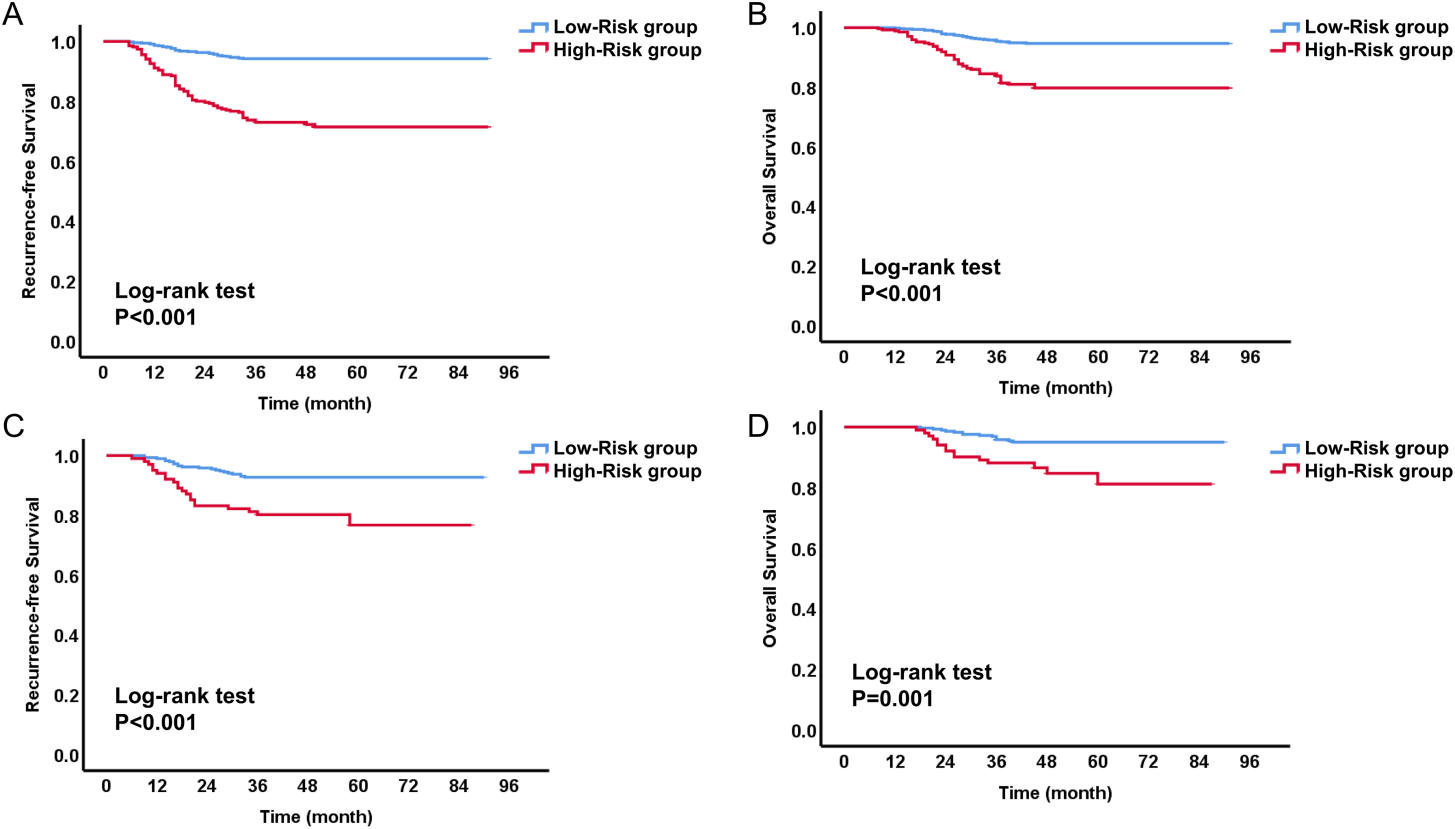
Kaplan–Meier survival curves for RFS and OS in the training cohort **(A, B)** and validation cohort **(C, D)**, comparing high-risk and low-risk groups stratified by the nomogram cutoff value.

The LNM probability for each patient in the training cohort was calculated using the model. Subsequently, the optimal cutoff value of the model for predicting LNM was determined by ROC curve analysis as 0.136 (AUC = 0.884, sensitivity = 80.1%, specificity = 81.2%) (**Figure 7**). Based on this cutoff, patients were stratified into a high-risk group (predicted probability ≥ 0.136) and a low-risk group (< 0.136). Kaplan–Meier survival analysis revealed significant differences in both RFS and OS among the high-risk and low-risk groups (all P < 0.001) (**Figure 8**; **Table 4**), suggesting that this model can effectively distinguish patient subgroups with distinct LNM risks. AUC-based comparison demonstrated that the nomogram developed in this study achieved superior predictive accuracy for LNM in EC patients, with AUC values of 0.889 in the training cohort and 0.866 in the validation cohort. This performance significantly outperformed Model A (29) (training: AUC 0.637; validation: AUC 0.617), Model B (30) (training: 0.703; validation: 0.659), and Model C (31) (training: 0.734; validation: 0.695) (P<0.05 for all comparisons; **Table 5**).

**Table 4:** Comparison of survival outcomes between high-risk and low-risk groups in training and validation cohorts.

Cohort	Group	LNM	Recurrence	3-year RFS (95%CI)	5-year RFS (95%CI)	P	Death	3-year OS (95%CI)	5-year OS (95%CI)	P
Training cohort (n=947)	High-risk group (n=271, 28.62%)	121(44.65%)	75(27.68%)	0.730 (0.677-0.783)	0.714 (0.657-0.771)	<0.001	53(19.56%)	0.838 (0.795-0.881)	0.797 (0.748-0.846)	<0.001
	Low-risk group (n=676, 71.38%)	30(4.40%)	39(5.77%)	0.942 (0.924-0.960)	0.942 (0.924-0.960)		35(5.18%)	0.954 (0.938-0.970)	0.947 (0.929-0.965)	
Validation cohort (n=389)	High-risk group (n=101, 25.96%)	38(37.62%)	21(20.79%)	0.802 (0.724-0.880)	0.767 (0.667-0.867)	<0.001	15(14.85%)	0.881 (0.818-0.944)	0.811 (0.711-0.911)	<0.001
	Low-risk group (n=288, 70.04%)	11(3.82%)	21(7.29%)	0.927 (0.898-0.956)	0.927 (0.898-0.956)		14(4.86%)	0.958 (0.934-0.982)	0.950 (0.925-0.975)	

CI, confidence interval; LNM, lymph node metastasis; OS, overall survival; RFS, recurrence-free survival.

**Table 5:** Comparative predictive performance of risk-stratification models for LNM in EC.

Model	Key predictors of the prediction model	Training cohort		Validation cohort	
		AUC(95%CI)	P	AUC(95%CI)	P
Model A^28^	Preoperative CA125 level	0.637 (0.586-0.689)	<0.001	0.617 (0.529-0.705)	<0.001
Model B^29^	Preoperative NLR and PLR	0.703 (0.654-0.752)	<0.001	0.659 (0.575-0.743)	<0.001
Model C^30^	Molecular classification	0.734 (0.691-0.778)	<0.001	0.695 (0.614-0.776)	<0.001
Our model	Preoperative age, CA125, histotype, molecular classification, and SIRI	0.889 (0.858-0.919)	–	0.866 (0.803-0.930)	–

AUC, area under the curve; CI, confidence interval; EC, endometrial cancer; LNM, lymph node metastasis; NLR, neutrophil-to-lymphocyte ratio; PLR, platelet-to-lymphocyte ratio; SIRI, systemic inflammatory response index.

## Discussion

LNM is a critical factor influencing the progression and prognosis of EC. Patients with pelvic or para-aortic LNM experience significantly worse outcomes compared to those without nodal involvement (18). Currently, there remains a lack of reliable preoperative models for stratifying the risk of LNM in EC patients. In this study, we developed a novel nomogram integrating key preoperative parameters, including age, serum CA125 level, histological type, molecular classification, and SIRI to predict LNM risk. Compared against traditional grading systems, our nomogram demonstrated superior performance in LNM risk stratification. This model provides a valuable tool to assist surgeons in selecting optimal surgical strategies, minimize surgical morbidity, and improve patient outcomes.

The multivariate logistic regression analysis confirmed that SIRI was an independent predictor of LNM in EC patients. This finding suggests that SIRI, derived from neutrophils, monocytes, and lymphocytes, provides a more comprehensive reflection of the systemic inflammatory burden and immune status, providing unique prognostic information beyond traditional parameters. The relationship between systemic inflammation and EC has been evaluated in recent years, particularly in terms of pre-treatment assessment and prognosis prediction (32). However, comparable to most biological markers, SIRI demonstrates insufficient predictive power when applied in isolation. Therefore, we further integrated SIRI with clinicopathological factors such as age, CA125, histological type, and molecular classification to construct a comprehensive nomogram prediction model. Internal and external validation demonstrated that this combined model exhibited good predictive accuracy. This nomogram tool allows clinicians to convert individual patient characteristics into a quantified total score, directly corresponding to the probability of LNM risk. For instance, a patient aged 70 years (39.5 points) with serum CA125 of 38 U/mL (50 points), histological type II (69 points), a SIRI score of 1.35 (≥ 1.115, categorized as high-risk: 100 points), and dMMR (37.5 points) would achieve a cumulative total risk score of 296 points, corresponding to an 86% predicted probability of LNM. Using the optimal cutoff of 0.136 (~139.5 points), patients are readily stratified into high- and low-risk groups. To facilitate clinical adoption, we propose a three-step workflow (1): Data acquisition—routine blood counts for SIRI and endometrial biopsy for histology plus MMR/p53 immunohistochemistry (2); Risk calculation—inputting variables into the nomogram; and (3) Clinical decision-making—scores <139.5 support SLNB alone or omission of extensive dissection to minimize morbidity, whereas scores ≥139.5 mandate systematic pelvic and/or para-aortic lymphadenectomy and proactive adjuvant therapy planning.

Multiple retrospective studies have shown that systemic inflammatory markers such as the NLR and SII are positively correlated with the risk of LNM. Patients with higher inflammatory burden generally demonstrate a higher probability of developing LNM, which is consistent with the findings of the present study (33, 34). The robust predictive value of SIRI for LNM can be explained by the complex interplay between systemic inflammation and the tumor microenvironment (TME). Elevated SIRI reflects a high proportion of neutrophils and monocytes alongside relative lymphopenia. Neutrophils can promote tumor invasion and angiogenesis by secreting vascular endothelial growth factor (VEGF) and matrix metalloproteinases (MMPs), which degrade the extracellular matrix and facilitate lymphatic spread (35, 36). Concurrently, circulating monocytes are recruited to the TME and differentiate into tumor-associated macrophages (TAMs) (37). TAMs are known to secrete immunosuppressive cytokines and growth factors that directly promote tumor cell migration and establish pre-metastatic niches in regional LN (38). Conversely, lymphocytes are key effectors of anti-tumor immunity. A relative decrease in lymphocytes indicates compromised host immune surveillance, allowing circulating tumor cells to evade destruction and metastasize (39). Therefore, SIRI acts as a comprehensive surrogate marker for this pro-metastatic, immunosuppressive state (40).

Notably, this study incorporated molecular classification into the LNM risk prediction system, fully considering the central regulatory role of molecular characteristics in EC progression and metastasis, which represents a key advantage of our predictive model. The EMSO and NCCN guidelines have both emphasized the prognostic importance of molecular markers in EC, highlighting their role in tailoring adjuvant therapy (41, 42). Instead of the widely accepted TCGA classification, this study adopted the ProMisE classification, dividing patients into three categories: pMMR-p53 wt, dMMR, and p53 abn. The feasibility of this classification has been demonstrated by multiple studies (43). A recent Brazilian cohort study further validated the ProMisE classifier, reporting a concordance rate of 86.8% between traditional and molecular approaches, with significant associations with OS and progression-free survival (44). Studies have demonstrated that the ProMisE classifier is a clinically practical classification scheme adapted from the TCGA classification system by Western researchers. By integrating MMR protein expression and p53 status for stratification without relying on high-throughput sequencing, it better aligns with the practical requirements of preoperative clinical testing. Moreover, ProMisE shows high concordance with the TCGA classification, effectively reflects the molecular heterogeneity of EC, and provides reliable evidence for clinical diagnosis and treatment decision-making. Constrained by preoperative testing conditions and the practical feasibility of clinical sample testing, this study adopted the ProMisE classification rather than the standard TCGA classification. Although this classification effectively incorporates molecular risk factors and meets the core need for preoperative LNM risk prediction, it does not capture the biological characteristics and differential metastatic risks of certain molecular subtypes (e.g., POLE-mutant) as finely as the TCGA classification, which may slightly affect the precision of the predictive model.

Furthermore, while several preoperative LNM prediction models have been proposed in recent years, our nomogram offers distinct clinical advantages. Beyond the conventional models evaluated in our study, contemporary research has largely focused on radiomics-based MRI models or extensive multi-gene sequencing panels (45–47). While radiomics models demonstrate high accuracy, they heavily rely on advanced imaging software, require substantial post-processing time, and are subject to inter-observer variability. Similarly, comprehensive genomic profiling remains cost-prohibitive and inaccessible in many resource-limited settings. In contrast, our model organically integrates standard clinicopathological data, an accessible 3-tier ProMisE molecular classifier based on routine immunohistochemistry, and SIRI, which is derived from a universal, low-cost preoperative CBC. This makes our nomogram highly scalable, cost-effective, and readily applicable in diverse clinical environments without the need for specialized equipment.

Nevertheless, this study has several limitations. First, this study was retrospective in design; prospective validation is needed to confirm the generalizability and robustness of our model. Second, the three-tier molecular classification used in this study did not capture the POLE ultramutated subtype. POLE testing was unavailable for 88.6% of the training cohort and 93.3% of the validation cohort due to the retrospective design and lack of insurance reimbursement. Because POLE−mutant tumors are characterized by an exceptionally low metastatic propensity, their misclassification into the pMMR/p53 wt group would artificially elevate the apparent LNM risk of that subgroup, introducing a directional bias. Future studies incorporating universal POLE testing and the complete four−tier TCGA/ProMisE classification are warranted to refine subgroup−specific risk estimation. Additionally, expanding the sample size and incorporating more comprehensive clinical and molecular data would further improve the accuracy, robustness, and clinical applicability of the predictive model, providing a more reliable tool for preoperative LNM risk stratification.

## Conclusions

In summary, this study identifies preoperative SIRI as an independent predictor of LNM in EC. The nomogram model developed based on preoperative SIRI and clinicopathological parameters enables individualized LNM risk stratification, helps identify high-risk patients who may be missed by traditional models, and provides a valuable tool for optimizing personalized management strategies.

## Data Availability

The raw data supporting the conclusions of this article will be made available by the authors, without undue reservation.
